# Thin SnO_x_ films for surface plasmon resonance enhanced ellipsometric gas sensing (SPREE)

**DOI:** 10.3762/bjnano.8.56

**Published:** 2017-02-28

**Authors:** Daniel Fischer, Andreas Hertwig, Uwe Beck, Volkmar Lohse, Detlef Negendank, Martin Kormunda, Norbert Esser

**Affiliations:** 1 BAM, Division 6.7, Surface Modification and Measurement Technique, Unter den Eichen 44–46, 12203 Berlin, Germany; 2 BAM, Division 2.1, Gases, Gasplants, Unter den Eichen 44–46, 12203 Berlin, Germany; 3 J.E.Purkinje University, Faculty of Science, Department of Physics, Ceske mladeze 8, 400 96 Usti nad Labem, Czech Republic; 4 Leibniz Institut für Analytische Wissenschaften ISAS e.V., Schwarzschildstr. 12, 12489 Berlin, Germany

**Keywords:** doped tin oxide, ellipsometry, gas sensing, surface plasmon resonance, thin films, transparent conductive oxides

## Abstract

**Background:** Gas sensors are very important in several fields like gas monitoring, safety and environmental applications. In this approach, a new gas sensing concept is investigated which combines the powerful adsorption probability of metal oxide conductive sensors (MOS) with an optical ellipsometric readout. This concept shows promising results to solve the problems of cross sensitivity of the MOS concept.

**Results:** Undoped tin oxide (SnO_x_) and iron doped tin oxide (Fe:SnO_x_) thin add-on films were prepared by magnetron sputtering on the top of the actual surface plasmon resonance (SPR) sensing gold layer. The films were tested for their sensitivity to several gas species in the surface plasmon resonance enhanced (SPREE) gas measurement. It was found that the undoped tin oxide (SnO_x_) shows higher sensitivities to propane (C_3_H_8_) then to carbon monoxide (CO). By using Fe:SnO_x_, this relation is inverted. This behavior was explained by a change of the amount of binding sites for CO in the layer due to this iron doping. For hydrogen (H_2_) no such relation was found but the sensing ability was identical for both layer materials. This observation was related to a different sensing mechanism for H_2_ which is driven by the diffusion into the layer instead of adsorption on the surface.

**Conclusion:** The gas sensing selectivity can be enhanced by tuning the properties of the thin film overcoating. A relation of the binding sites in the doped and undoped SnO_x_ films and the gas sensing abilities for CO and C_3_H_8_ was found. This could open the path for optimized gas sensing devices with different coated SPREE sensors.

## Introduction

Gas sensors are an important tool for example in the fields of process monitoring, workplace safety or environmental analysis. Due to the wide field of possible applications, many different concepts for gas sensing were developed in the past. Typical state-of-the-art concepts for gas measurements are electrochemical and infrared sensors, pellistors and metal oxide conductive sensors (MOS) [1]. All sensing concepts have their respective properties. Electrochemical sensors are limited to several gas species [2]. The infrared (IR) sensor needs a certain optical path for the IR beam inside the gas volume but shows high sensitivity and selectivity [3]. Pellistors can only measure specific gases due to their catalytic combustion concept but are fast and accurate [4]. Conductive MOS sensors show sensitivities to the ppb range but have high cross sensitivities to other gases [5]. These sensors detect gases by measuring resistance changes due to the adsorption of gas molecules on the surface [6–7]. This surface enhanced and adsorption driven concept has a potential for a wide range of applications because it depends on the gas–surface interaction. This dependence enables us to tune the adsorption properties for specific gases by using different preparation techniques or introduce doped thin films. In the last years, the use of tin oxide (SnO_x_) layers for gas sensors has attracted some interest [8–10]. These layers are widely used due to their excellent performance in the detection of gases. Due to the high dependency of the sensitivity of these layers in the gas detection on the preparation procedure, several different coating methods were developed which include chemical vapor deposition [11], sol–gel [12], spray pyrolysis [13], sputtering [14–16] and electron beam evaporation [17]. In our approach, we aim to develop a new sensing concept which combines the adsorption concept of MOS sensors with optical ellipsometric readout from the backside which means that the probing laser light does not need a pathway inside the gas volume. Additionally, the cross sensing problems can be solved by modifications enhancing the sensing probability for only one gas species. The aim of this study is a deeper understanding of the relation of the overcoating tin oxide layers and their gas sensing abilities.

## Methods

### Experimental

The sensors were prepared by a combination of two different techniques which were already reported [15–16]. The uncoated rectangular substrate prisms are made of N-BK7 glass with a leg length of 7 mm (Edmund Optics). The prisms were coated on the hypotenuse face with a 45 nm gold layer by using the electron beam evaporation technique (CS 730 ECS, von Ardenne Anlagentechnik GmbH). The additional undoped SnO_x_ add-on layer was then added by using radio frequency (RF) magnetron sputtering (CS 730 ECS, von Ardenne Anlagentechnik GmbH) with 13.56 MHz frequency and 200 W power. Here, a commercially available pure SnO_2_ target (99.9%) obtained from FHR GmbH was used. The target has a diameter of 200 mm and a thickness of 6 mm. For the Fe-doped samples, a home-built RF magnetron sputtering equipment with 13.56 MHz frequency was used with a SnO_2_ target (99.95%) of 48 mm diameter. In this case, an additional rectangular Fe-strip (99.95%) of size 10 mm × 20 mm and a thickness of 2 mm was attached at the center of the SnO_2_ target. The deposition was done by applying 50 W of DC pulsed power at a frequency of 50 kHz with a pulse duration of 4 μs. Depending on the intended layer properties, the parameters of the sputtering were changed. These parameters are the partial pressure of argon and (if added) oxygen, bias and deposition time. For the undoped SnO_x_ layers, pure argon with a pressure of 0.3 Pa was used. The Fe-doped SnO_x_ was deposited with a 20%/80% oxygen/argon mixture with a pressure of 0.2 Pa. The thickness of the SnO_x_ and Fe:SnO_x_ overcoatings was 7 nm and 5 nm, respectively. The thickness was determined by using spectroscopic ellipsometry. The layer morphology was also determined by means of atomic force microscopy (AFM) and transimission electron mircoscopy (TEM) measurements. These measurements are reported in [18–19]. It was found that the intended layer structure (glass-gold-SnO_x_) was achieved and the surface roughness is low (root mean square average of height deviation *R*_q_ < 0.2 nm). Due to the low *R*_q_ value, roughness effects were not taken into account for explanation of the results. Furthermore, no indication of changes in the porosity of the layers which probably could effect the gas sensing ability was found.

**Figure 1:**
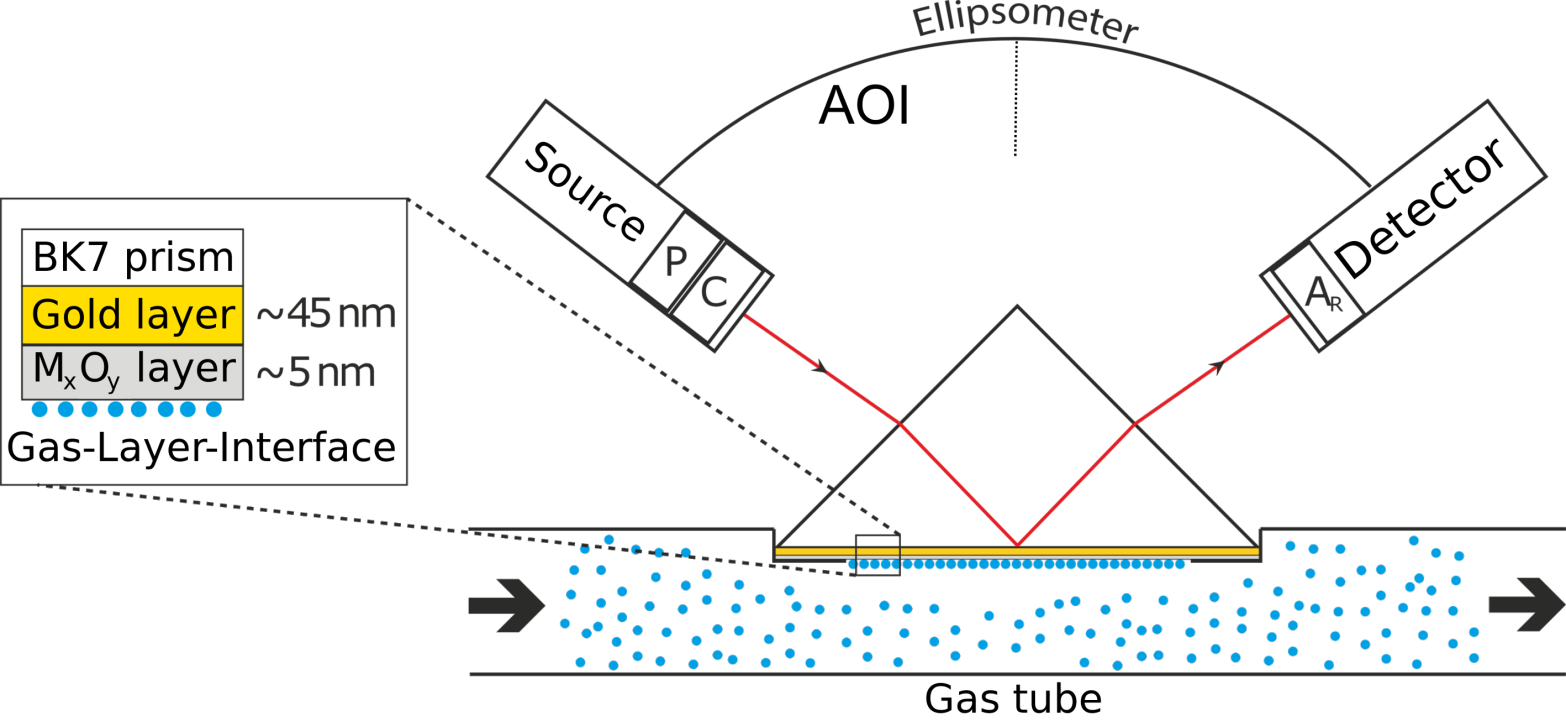
Scheme of the SPREE gas sensing device.

The gas sensing technique called surface plasmon resonance enhanced ellipsometry (SPREE) has been described previously [20–22]. The scheme of the device is shown in Figure 1. It consists of the sensing unit with the prepared layer system which is placed onto a gas tube which was prepared with a hole. The gas tube is filled with a gas mixture consisting of synthetic air (20% O_2_, 80% N_2_) and one added analyte gas (CO, H_2_ or C_3_H_8_). The analyte gas was mixed with synthetic air by using a home-built mixing setup which is regulated by a MKS 647 C multi gas controller monitor. The flow was kept constant at 100 sccm. The pressure inside the gas tube was manipulated by using a high-precision valve (SS-6BMG-MM, Swagelok). The unit of sensor and attached gas tube is placed into the measurement spot of a single wavelength laser ellipsometer (Sentech SE 400). The ellipsometer is equipped with a helium–neon laser source (632.8 nm), a polarizer (P), a switchable prism compensator (C) and a rotating analyzer (A_R_). The angle of incidence (AOI) can be changed by means of a manual goniometer with a precision of 0.05°.

### Surface plasmon resonance enhanced ellipsometry

A theoretical description of the SPREE technique is given in detail elsewhere [23–25]. In ellipsometry, the complex reflectance ratio ρ after irradiation of a sample with linear polarized light is measured. For a simple two-media dielectric interface, the complex reflectance ratio is given by [26–27]

[1]
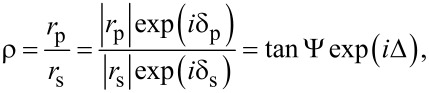


with *r*_p,s_ as the Fresnel coefficent and δ_p,s_ as the phase for the p- and s-polarization state. tanΨ and Δ are the consecutive amplitude ratio and phase difference of ρ. If a thin semitransparent layer like a gold layer is introduced between the two dielectric media, the overall reflection coefficient will change to

[2]
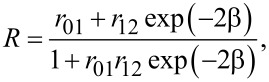


with *r*_01_ as the Fresnel coefficient at the interface of the incident layer (BK7 glass) and the thin gold and *r*_12_ as the interface between the gold layer and the dielectric on the bottom side of the setup (air). The β value describes the film phase thickness given by the formula

[3]
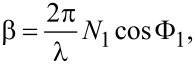


with *N*_1_ as the complex refractive index of medium 1 und Φ_1_ as the incident light angle in medium 1.

**Figure 2:**
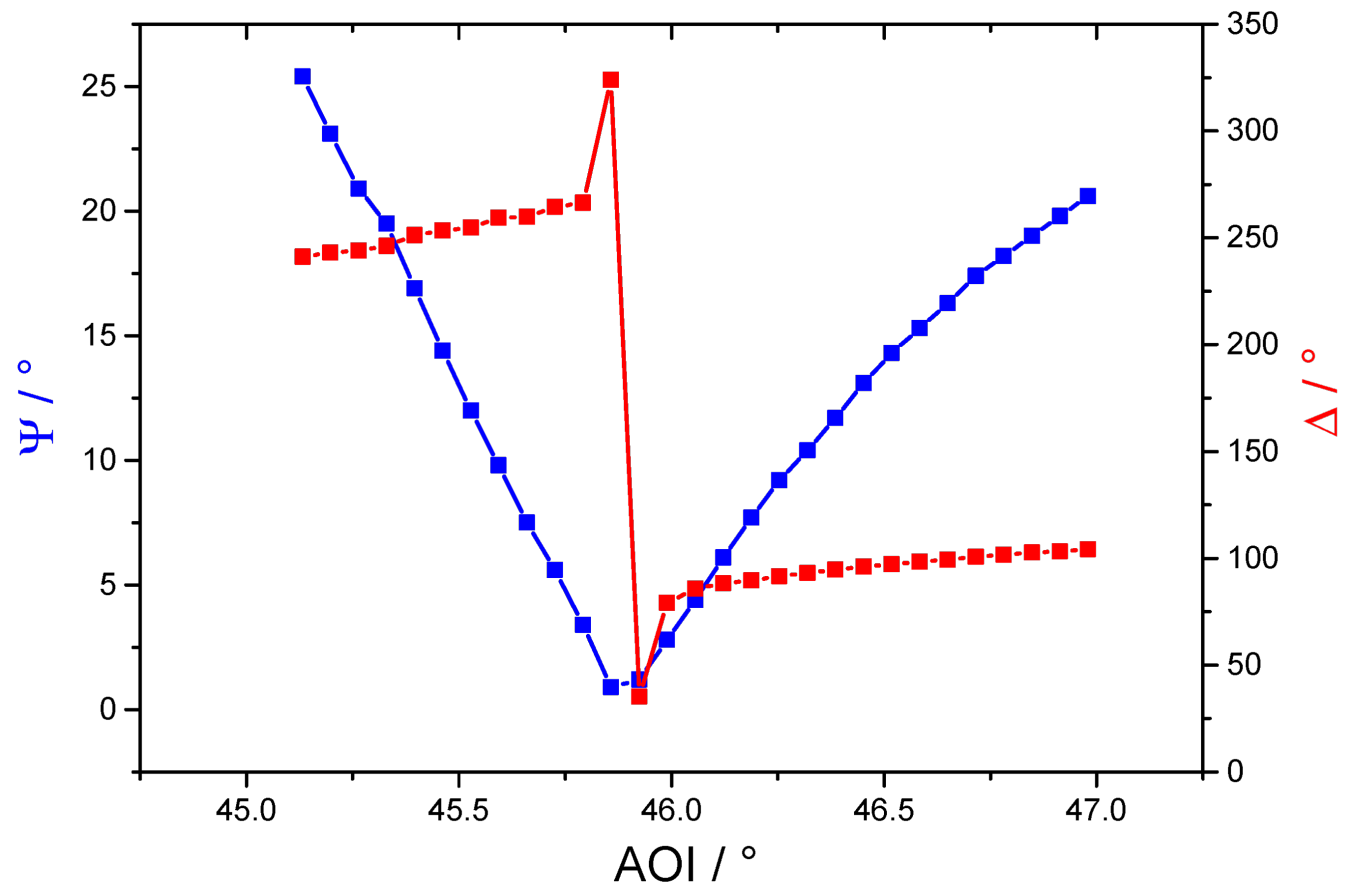
Measured data of Ψ and Δ for a BK7 glass prism with a 45 nm Au coating and a 5 nm SnO_x_ add-on layer.

By knowing that the intermediate layer is gold, the effect of the surface plasmon resonance has to be taken into account for the description of the reflection coefficients. Because this phenomenon effects mostly the reflection coefficient of the p-polarized part *R*_p_, the SPR effect can be described by the reflectance 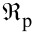 = |*R*_p_|^2^. Close to the surface plasmon resonance, the reflectance can be written as

[4]
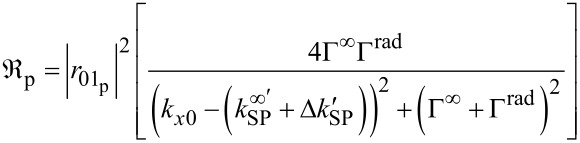


with *k_x0_* as the component of the wave vector of the incident light in the direction of the surface plasmon propagation and *k_SP_* = 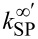 + *i*Γ^∞^ as the wave vector of a surface plasmon on an interface of two semi-infinite media according to [28]. Due to the finite thickness of the gold film, a perturbation term is introduced by using 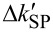 + *i*Γ^rad^. In the ellipsometric measurement the Ψ and Δ values can be interpreted by [23–25,28].

[5]
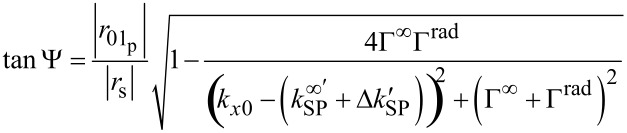


[6]
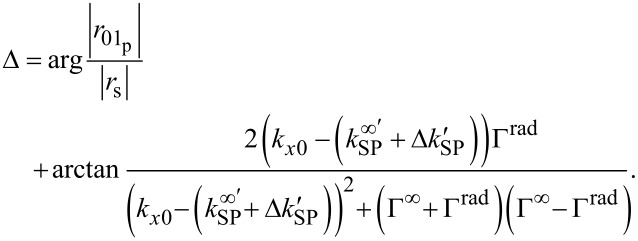


If the angle of incidence is varied close to the resonance condition, the Ψ function shows a minimum at the exact resonance angle, while in the Δ graph a step function can be observed. Both values reach their minimum due to the complete coupling to the plasmon resonance if an ideal system is assumed. If an additional layer is introduced between the gold layer and the ambient dielectric medium (air), the simulation of the system becomes more complex. In this case an extra pertubation has to be taken into account which will disturb the ideal resonance conditions. This case is shown in Figure 2, where a 5 nm thick tin oxide layer was added to the system. This perturbation leads to a shift of both functions to a higher resonance angle as well as an increased minimum of Ψ and Δ.

### Application of the SPR theory to gas sensing

By changing the dielectric medium (air) with an addition of a certain volume of another gas, a shift in the dependence of Ψ and Δ on the AOI is predicted. The reason of this change can be explainend by different effects. First, a change of the refractive index of the dielectric medium (air) due to addition of another gas can be considered. In this case, the new refractive index of the gas mixture is given by the Lorenz–Lorentz-equation [29]

[7]
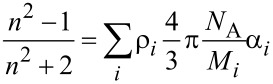


with ρ*_i_* as the partial density of the *i*th component, *N*_A_ as the Avogadros constant, *M_i_* as the molecular weight and α*_i_* as the polarizability. By using the changed refractive index of the gas mixture, the values of Ψ and Δ in the SPREE experiment can be calculated using Equation 5 and Equation 6. However, as the partial density of the added gas is in the dimension of 10^−6^ (ppm), this theory does not explain the measured changes in Ψ and Δ as presented in this study because the quantitative change would be immeasurably small. To explain this sensitivity, the adsorption of molecules on the surface of the sensor has to be taken into account. In this case, as known from SPR spectroscopy [30–31], a new thin layer of adsorbed molecules with the dimension of a few nanometer thickness will change the position of the surface plasmon resonance peak in the angular spectrum to higher angles. This effect depends on the thickness and the refractive index of the layer of adsorbed molecules. In the case of a gas mixture with one gas component added, the thickness of the adsorbed layer is adjusted in correlation with the concentration of this gas in the mixture. If the amount of adsorbed molecules at the surface can be increased, e.g., by providing additional binding sites using a doped overcoating or by enhancing the porosity of the layer, the sensitivity of the gas measurement will be raised. Additionally, the pressure of the gas volume will have an effect to the measured signal because it is proportional to the amount of adsorbed molecules on the surface and because the refracive index of the gas phase rises with the rising pressure.

## Results and Discussion

### Pressure dependence

**Figure 3:**
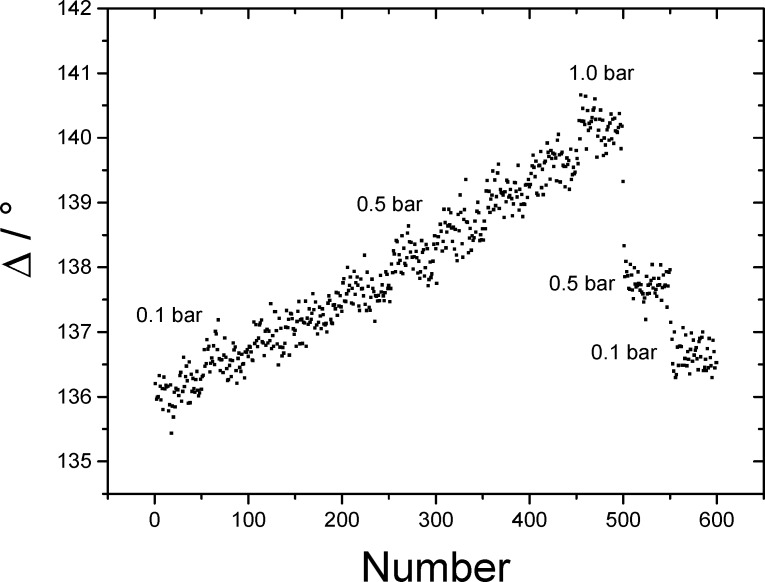
Δ signal depending on different air pressure values at an AOI of 47.9°. The pressure was changed every 50 measurement points by 0.1 bar up to a pressure of 1.0 bar. Then the pressure was reduced to 0.5 and 0.1 bar.

As described above, the gas experiments show a pressure dependence, which changes the Δ response. By changing the relative pressure inside the gas tube, a signal could be detected with the SPREE experiment. The resulting signal in dependence to the relative pressure is shown in Figure 3. The response was measured with steps of 0.1 bar. The pressure was measured by using a gauge relative to atmospheric pressure.

This pressure dependence can be a result of the changed amount of molecules adsorbed to the surface which effect the position of the SPR signal. Another explanation could be a changed refractive index of the gas medium due to the different density by increasing the pressure. This behavior shows a linear dependency in the studied pressure region to the pressure which is in agreement with both explanations. Supposedly, a combination of both mechanisms occurs when the total pressure of the gas phase is changed. To achieve the linearity and sensitivity shown in the following gas measurement section, it is necessary to keep the pressure constant during the experiment. Otherwise this additional degree of freedom will result in unpredictable response of the Δ signal to changes in the gas tube. We solved this by using a high precision pressure valve which is adjusted in dependence of the pressure gauge to keep the pressure constant. The pressure value for all gas experiments was adjusted to be 0.4 bar overpressure compared to ambient.

### Gas sensing experiments

**Figure 4:**
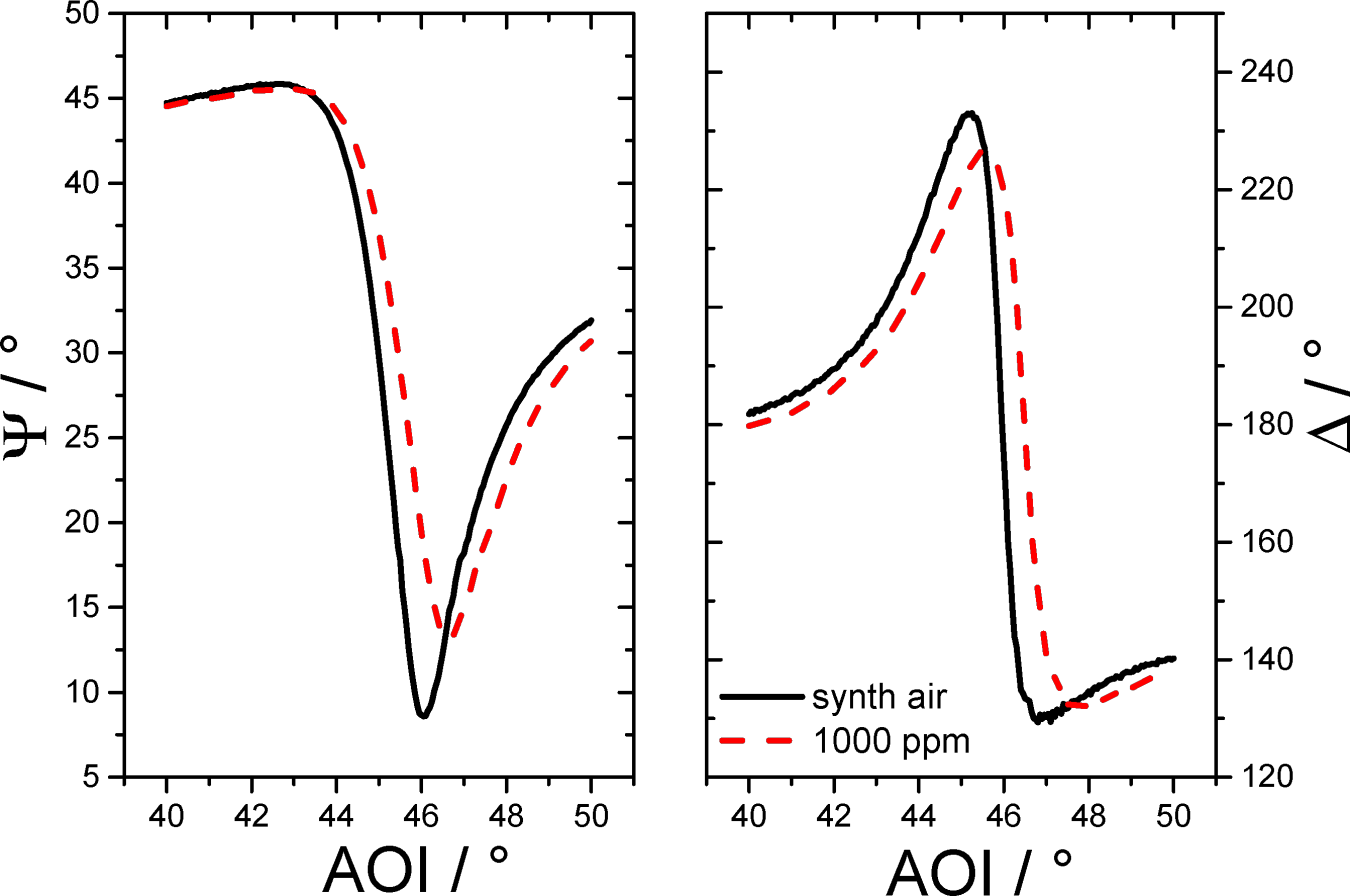
Changes in the Ψ and Δ angle spectrum due to changing gas atmosphere. Black / solid: synthetic air. Red/dashed 1000 ppm CO in synthetic air.

A typical gas sensing experiment is shown in Figure 4 where the gas was changed from synthetic air to a mixture of 1000 ppm CO in synthetic air.

**Figure 5:**
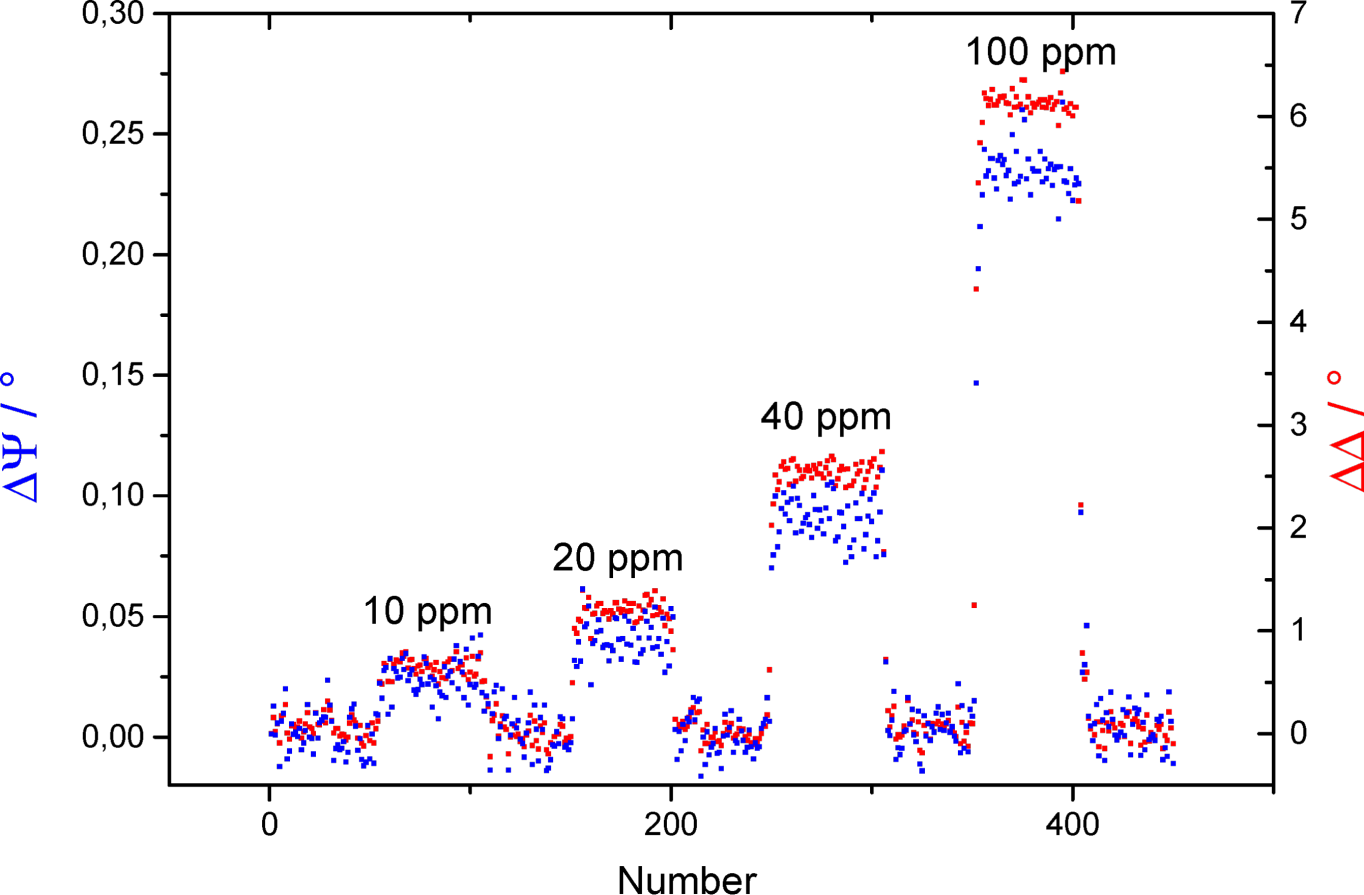
Comparison of the change in Ψ and Δ at an AOI of 44.5° after exposure for different concentrations of CO on a Fe:SnO_x_ coating. The standard deviations σ for ΔΨ and ΔΔ are 0.04° and 0.016°, respectively.

Here, a shift of 0.5° of the Ψ minimum and the Δ phase change can be observed on the AOI axis. The amount of this shift is related to the change in frequency of the surface plasmon resonance due to the adsorption of the gas molecules on the surface as described above. If the response of the Ψ and Δ signal is monitored over a certain time while the gas concentration is changed, a difference of the signal is measured as shown in Figure 5. The ΔΔ value (same for ΔΨ) shows the change of the ellipsometric Δ value after exposure with a analyte gas concentration in relation to the situation without gas exposure (ΔΔ = 0). This nomenclature was chosen because only the change of the ellipsometric quantities is relevant for the gas measurement.

**Figure 6:**
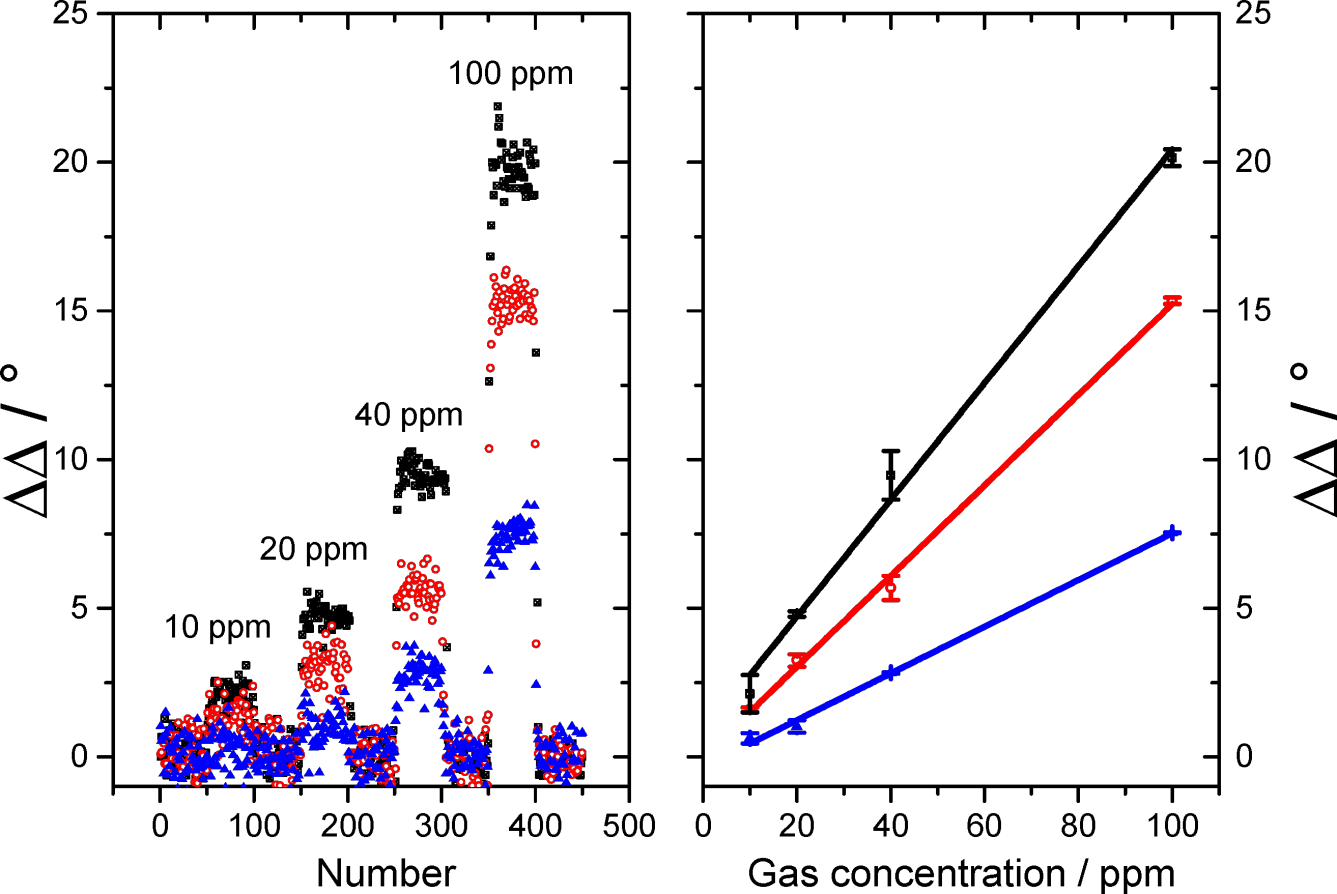
Gas measurement of C_3_H_8_ (black rectangle), CO (blue triangle) and H_2_ (red dots) with SPREE with an undoped SnO_x_ layer at an AOI of 45.3° (left). Linearity analysis of the ΔΔ response to the gas concentration (right).

**Figure 7:**
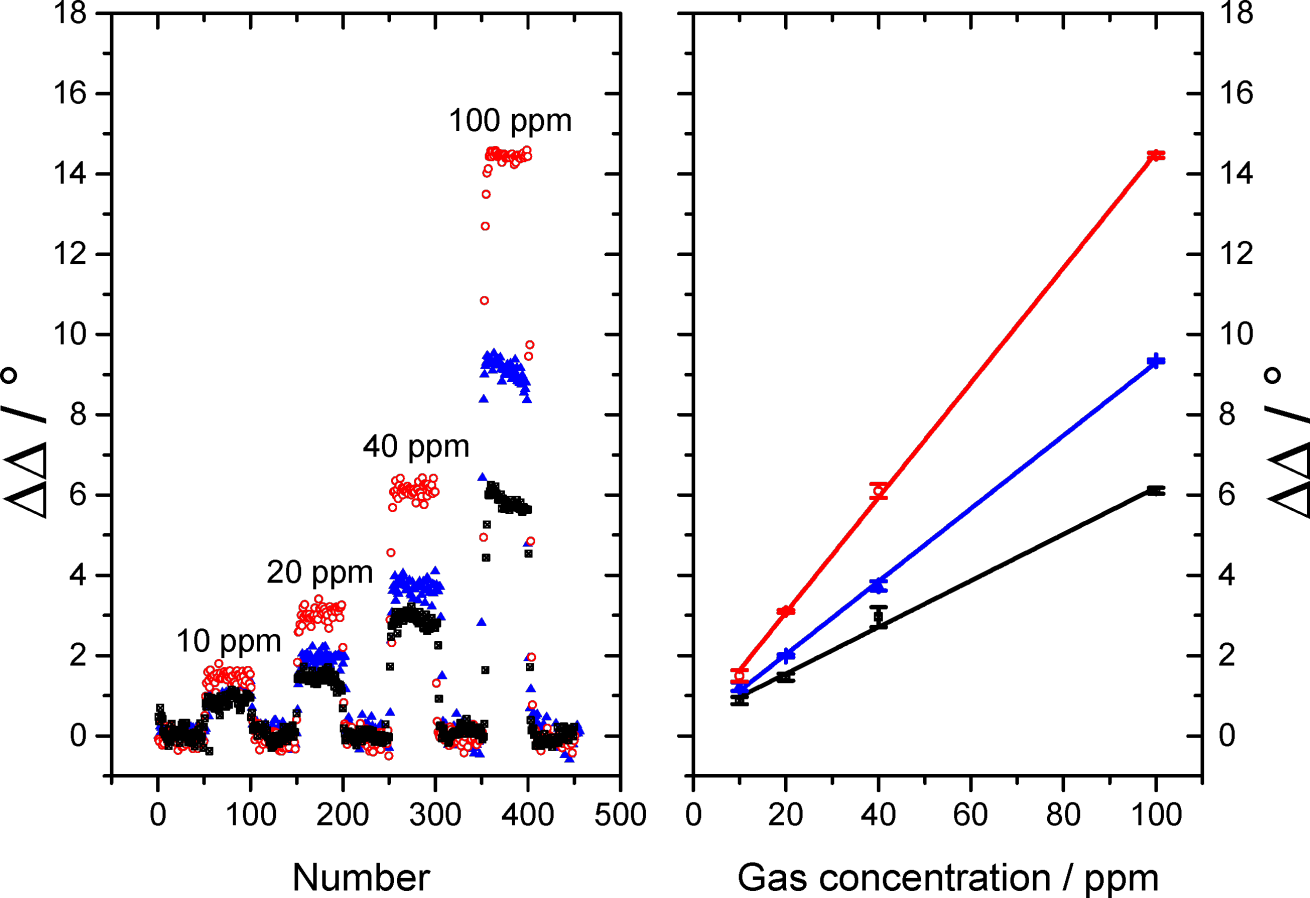
Gas measurement of C_3_H_8_ (black rectangle), CO (blue triangle) and H_2_ (red dots) with SPREE at an AOI of 44.5° with an Fe:SnO_x_ layer (left). Linearity analysis of the ΔΔ response to the gas concentration (right).

We decided to focus on the evaluation of the ΔΔ values instead of analyzing the ΔΨ values because the signal to noise ratio is much better for the ΔΔ measurement. The calculated values of the standard deviation σ for the gas measurement of CO on a Fe:SnO_x_ overlayer, as shown in Figure 5, are 0.04° for ΔΨ and 0.016° for ΔΔ, respectively. This lower σ value leads to a better signal to noise ratio and, consequently, a higher sensitivity in the gas measurement. The measurement of three different gases is shown in Figure 6 and Figure 7 with ΔΔ as the difference in Δ over the total number of measurements. Each of them represents a ellipsometric measurement and takes approximately 3 s.

It was found that all three gases, C_3_H_8_, CO and H_2_, can be measured down to the low ppm range with different sensitivity. All gases show a fast response to concentration changes. It takes approximately 3–4 measurements to reach the plateau of the signal which represents 9–12 s response time. This time is likely to be dominated by the time needed for the gas mixture to establish in the apparatus. Therefore, we suppose that all surface processes generating the measurement effect are faster than the time resolution of the experiment. All changes are fully reversible and the signal regenerates to the initial value within the same time of 9–12 s. As shown in Figure 6 and Figure 7, the signal shows different intensities depending on the gas species and layer type. For the undoped SnO_x_ add-on layer shown in Figure 6, C_3_H_8_ shows the highest signal of 20° in ΔΔ for 100 ppm and is well resolved at 10 ppm with 2.2°. For H_2_ the maximum signal for 100 ppm reaches ΔΔ = 15° and can only barely identified at 10 ppm with ΔΔ = 1.5°. The lowest response was detected for CO with ΔΔ = 7.5° at 100 ppm and almost no visible signal at 10 ppm.

All signals respond linearly to concentration changes as plotted on the right graph in Figure 6. This linearity is a very important aspect of this method for future applications. The different sensitivities to the three measured gases can be explained by the different adsorption behavior of each gas on the surface. By using an undoped SnO_x_ layer as the overcoating for the gold sensing layer, the adsorption probability of the sensor layer system is changed. The undoped SnO_x_ coating causes a stronger response of the sensor signal for C_3_H_8_ as for CO. This observation supports the assumption that C_3_H_8_ is more likely adsorbed than CO.In contrast, the response to H_2_ is driven by an increased diffusion into the sensing layer in contrast to an adsorption at the surface due to the small molecule size [32]. To support the theory of a dependence of the binding sites to the response in the gas measurement, iron doped tin oxide (Fe:SnO_x_) overcoated sensors with an increased number of binding sites for CO were built [33]. This was achieved by adding a block of metallic iron to the target. A gas sensing measurement using a SPR setup with this coating is shown in Figure 7. By using this type of coating, the response to the gas species and the overall sensitivity has changed. The best response was achieved for H_2_ with 14.5° for 100 ppm which is comparable with the response of the undoped SnO_x_ layer analyzed in the prior experiment. As the response of H_2_ is the same for undoped and Fe-doped SnO_x_, the assumption that the sensing mechanism is not based on adsorption of the molecule on the surface but on a diffusion process into the sensing layer is supported for this gas species. The signal for 10 ppm is still well resolved with ΔΔ = 1.6°. The better resolution in comparison to the undoped sample is a result of the much better signal to noise ratio achieved with the Fe:SnO_x_ add-on layer. This effect is probably due to a changed thickness (5 nm for Fe:SnO_x_ and 7 nm for SnO_x_) of the layer which also effects the distinct sharp form of the SPR signal. In another study, we have found that the thickness of the overlayer affects the gas sensing sensitivity but not the selectivity to a specific gas species. By tuning the thickness to a certain limit (depending on the metal oxide system), the ability to measure a lower concentration of the added gas species is improved [18]. The second best response was achieved for CO gas with a value of ΔΔ = 9.5° for 100 ppm and a measurable resolution at 10 ppm with ΔΔ = 1.2°. The response is slightly better than in the prior experiment which was ascribed to the higher amount of adsorbed molecules due to increased number of binding sites in the add-on layer. The lowest signal was determined for C_3_H_8_ gas with ΔΔ = 6.1° at 100 ppm and ΔΔ = 1.0° at 10 ppm. This extensive decrease in the response for C_3_H_8_ in comparison to the undoped SnO_x_ coating corroborates the theory of a reduction of the amount of adsorbed molecules due the raised number of binding sites for CO.

## Conclusion

The recently developed gas measurement technique, surface plasmon resonance enhanced ellipsometry (SPREE), was investigated in terms of sensitivity, reversibility and linearity. Additionally, the relation between changes of the dielectric function due to changes of the gas species and concentration was investigated. It was found that the gas measurement with all probed gas species (H_2_, CO and C_3_H_8_) is fully reversible even after several cycles. Furthermore, a linear response to the concentration of every gas species was found which shows a promising behavior for future applications. Further investigations to optimize the sensitivity for specific gas species have shown that the response of the ellipsometric Δ signal depends on the overcoating of the actual gold sensing layer. Two different add-on layers were tested, undoped SnO_x_ and iron doped SnO_x_ (Fe:SnO_x_) due to their different amount of binding sites for C_3_H_8_ and CO. The assumed effect that an additional amount of binding sites for CO, as present in Fe:SnO_x_, will lead to an increased sensing ability for CO is supported by the results. In consequence, a decreased signal for C_3_H_8_ was found. No effect in dependence of the doped layer was observed for H_2_. This was explained by a different sensing mechanism which does not rely on the adsorption of the molecules on the surface but on the diffusion into the sensing layer. However, the detailed sensing mechanism could not be determined due to the lack of temperature dependent measurements. An additional linear dependence to the gas pressure was observed which needs to be controlled during the gas measurement to obtain reliable data on the dependence of the measurement signal on the additional layer. The new type of gas sensing with the SPREE method showed promising results by confirming that the sensitivity to a specific gas can be controlled by changing the add-on coating of the sensing gold layer. Additional studies investigating the influence of the add-on layer properties to the gas sensitivity and selectivity will follow.
